# Correspondence

**Published:** 1880-09

**Authors:** J. H. Warner

**Affiliations:** Columbus, O.


### CORRESPONDENCE.

Columbus, O., August 2, 1880.

Editor Register—In the August number of the Register I notice a statement made by a gentleman of Tennessee, which is so fallacious that to simply call attention to it is to controvert it. He says: "It is estimated that there are in this country alone 12,000 dentists, and the annual consumption of pure gold made into foil for this country alone will amount to three tons, costing over $3,000,000." Now, three tons is 60,000 pounds—an average of five pounds to each dentist per year—which, at twelve ounces to the pound, is sixty ounces, costing, at $30 per ounce, $1,800 per year for each dentist for gold foil alone; or, reckoning 300 days to the year, would be one book and six-tenths to each dentist daily.

Clearly the estimate is much too high, and if divided by five will be much nearer right. J. H. WARNER, D. D. S.

